# Metformin as an Adjunct to Fertility-Sparing Treatment in Atypical Endometrial Hyperplasia and Early-Stage Endometrioid Endometrial Cancer: Narrative Review

**DOI:** 10.3390/cancers18193141

**Published:** 2026-09-28

**Authors:** Angel Yordanov, Eva Tsoneva, Stoyan Kostov, Vesselina Yanachkova, Zlatko Kirovakov

**Affiliations:** 1 Department of Gynecologic Oncology, Medical University–Pleven, 5800 Pleven, Bulgaria; 2 Department of Gynecology, Military Medical Academy, 1606 Sofia, Bulgaria; dretsoneva@gmail.com; 3 Research Institute, Medical University–Pleven, 5800 Pleven, Bulgaria; drstoqn.kostov@gmail.com (S.K.); v_ess@abv.bg (V.Y.); 4 Department of Gynecology, St. Anna University Hospital, 9002 Varna, Bulgaria; 5 Department of Endocrinology, Specialized Hospital for Active Treatment of Obstetrics and Gynaecology “Dr Shterev”, 1330 Sofia, Bulgaria; 6 Faculty of Public Health and Health Care, Prof. Dr. Assen Zlatarov University, 8000 Burgas, Bulgaria; kirovakov@yahoo.com

**Keywords:** endometrial cancer, atypical endometrial hyperplasia, fertility preservation, metformin, progestin therapy, LNG-IUS, reproductive outcomes

## Abstract

**Objective:** This study aims to evaluate the potential role of metformin as an adjunct to fertility-sparing treatment strategies for women with atypical endometrial hyperplasia/endometrial intraepithelial neoplasia (AEH/EIN) and early-stage endometrioid endometrial cancer (EEC), with particular emphasis on oncologic and reproductive outcomes. **Methods:** A systematic search of PubMed/MEDLINE, Embase, Scopus, Web of Science, and the Cochrane Library was conducted from database inception through May 2026. Eligible studies included randomized controlled trials, prospective studies, and retrospective cohort studies evaluating metformin-containing fertility-sparing treatment regimens in women with AEH/EIN or early-stage EEC. Owing to substantial clinical and methodological heterogeneity in study design, treatment protocols, metformin dosage, follow-up duration, and outcome reporting, a quantitative meta-analysis was considered inappropriate, and the findings were synthesized narratively. **Results:** Eight publications represented approximately 795 unique patients after accounting for overlapping cohorts. Complete response was reported in 63/92 patients overall (69%) in Acosta-Torres et al., including 23/34 (68%) receiving progestin plus metformin, and in 61/63 (97%) receiving medroxyprogesterone acetate plus metformin in Mitsuhashi et al. Reported recurrence rates ranged from 13.1% to 34.2%. Live births occurred in 2/34 (6%) metformin-treated patients in Acosta-Torres et al., 14/31 (45%) women wished to conceive in Mitsuhashi et al., and 29/79 (36.7%) patients overall in Jing et al. Differences in populations, assessment times, and reproductive denominators limited direct comparisons and attribution of benefit to metformin. **Conclusions:** Current evidence suggests that metformin may have a promising adjunctive role in fertility-sparing treatment for women with AEH/EIN and early-stage EEC, particularly among patients with obesity, insulin resistance, polycystic ovary syndrome, or other metabolic risk factors. Nevertheless, the overall body of evidence should be interpreted with caution because of substantial heterogeneity across studies, the predominance of retrospective designs, and the limited number of prospective randomized trials. Well-designed multicenter randomized controlled trials are required to define the optimal role of metformin, identify the patients most likely to benefit, and establish standardized treatment strategies before routine incorporation into clinical practice can be recommended.

## Simple Summary

Fertility preservation is an important consideration for younger women with atypical endometrial hyperplasia or early-stage endometrial cancer, as standard surgical treatment involves removal of the uterus and therefore prevents future pregnancy. Metformin has been proposed as an additional treatment alongside hormone-based fertility-sparing therapy, particularly in women with obesity, insulin resistance, or other metabolic risk factors. This systematic review evaluates the available clinical evidence on whether metformin-containing treatment strategies may improve cancer-related and reproductive outcomes. Current findings suggest a potential beneficial role for metformin in selected patients; however, differences in study design, treatment approaches, and outcome reporting, together with the limited number of prospective trials, prevent definitive conclusions. By identifying these limitations and gaps in the evidence, this review may help guide future clinical research aimed at determining which patients are most likely to benefit and at developing more standardized fertility-sparing treatment strategies.

## 1. Introduction

Endometrial carcinoma is one of the most common malignancies affecting women, and its incidence is increasing in high-income countries [1], particularly among postmenopausal women [2]. Globally, up to 300,000 new cases of endometrial cancer are diagnosed annually [3,4], making it the fifth most common cancer in women. Its increasing prevalence has been attributed to obesity, metabolic syndrome, and other lifestyle-related risk factors [5,6]. A large proportion of endometrial cancers are endometrioid carcinomas, which develop along a continuum of endometrial abnormalities beginning with endometrial hyperplasia and progressing to malignant transformation in susceptible individuals [7,8]. These tumors are generally associated with estrogen-dependent pathogenesis [9]. Atypical endometrial hyperplasia (AEH) is a common premalignant lesion characterized by glandular crowding and cytological atypia; it carries a substantial risk of concurrent endometrial carcinoma or progression to invasive cancer if left untreated [10,11]. Although the disease predominantly affects postmenopausal women, its increasing incidence among younger women of reproductive age has important implications for fertility preservation and long-term reproductive health. Definitive management has traditionally relied on hysterectomy, which effectively eliminates the risk of disease progression but permanently compromises fertility. Consequently, fertility-preserving therapeutic approaches must be carefully evaluated to optimize both oncologic and reproductive outcomes.

Fertility preservation is now a major consideration in the management of atypical endometrial hyperplasia and early-stage endometrioid endometrial cancer. Fertility-sparing management is primarily based on progestin therapy. Oral progestins, such as medroxyprogesterone acetate and megestrol acetate, are standard treatment options because they counteract estrogen-driven endometrial proliferation and induce disease regression [12,13,14]. The levonorgestrel-releasing intrauterine system (LNG-IUS) has also emerged as an effective treatment option that delivers high local concentrations of progestin directly to the endometrium while minimizing systemic adverse effects [15,16]. Nevertheless, fertility-sparing therapies have important limitations, including progesterone resistance, in which patients exhibit a suboptimal or absent response to progestin therapy owing to alterations in progesterone receptor signaling, inflammatory pathways, and metabolic regulation [17,18]. Emerging evidence also suggests that obesity, insulin resistance, type 2 diabetes mellitus, and other components of metabolic syndrome may further reduce the effectiveness of standard therapies [19,20]. Given the strong association between metabolic abnormalities and endometrial neoplasia, metformin has emerged as a potential therapeutic intervention for patients with AEH and EEC. By improving insulin sensitivity and reducing circulating insulin levels, metformin may mitigate the proliferative effects of hyperinsulinemia on endometrial tissue. However, its role in fertility-sparing treatment remains incompletely defined. Accordingly, this systematic review evaluates metformin in the fertility-sparing management of AEH/EIN and early-stage EEC by synthesizing current evidence on oncologic outcomes, including treatment response and recurrence, and reproductive outcomes, including pregnancy and live birth rates.

Although interest in metformin as an adjunct to fertility-sparing treatment has increased in recent years, the available clinical evidence remains limited and heterogeneous. Therefore, an updated systematic review was undertaken to critically evaluate the current evidence regarding the oncologic and reproductive outcomes associated with metformin in women with AEH/EIN and early-stage EEC.

## 2. Methods

### 2.1. Study Design

This systematic review assessed the role of metformin in the fertility-sparing treatment of atypical endometrial hyperplasia/endometrial intraepithelial neoplasia (AEH/EIN) and early-stage endometrioid endometrial cancer (EEC), with a focus on oncologic and reproductive outcomes. The review was conducted and reported in accordance with the Preferred Reporting Items for Systematic Reviews and Meta-Analyses (PRISMA 2020) statement. The completed PRISMA 2020 checklist is provided in Supplementary Table S1, and the PRISMA 2020 flow diagram is presented in Figure 1. The review protocol was not prospectively registered in PROSPERO or another registry. However, before the literature search and study selection were conducted, the authors defined an a priori methodological approach that included the review question, eligibility criteria, databases and search concepts, study-selection procedures, outcomes of interest, data-extraction methods, and methodological quality assessment. These elements did not change during the review process, thereby minimizing the risks of protocol deviation and selective reporting. The absence of prospective protocol registration is acknowledged as a methodological limitation. Given the considerable clinical, methodological, and outcome heterogeneity across the included studies, a quantitative meta-analysis was deemed inappropriate. Clinical heterogeneity included differences in patient populations (AEH/EIN versus early-stage EEC), fertility-sparing strategies, metformin dosage and duration, concomitant hormonal therapies, and follow-up duration. Methodological heterogeneity reflected differences in study design, including randomized, prospective, and retrospective studies. Outcome heterogeneity arose from variation in outcome definitions, the timing of response assessment, biopsy and surveillance protocols, and the reporting of oncologic and reproductive outcomes. Collectively, these sources of heterogeneity precluded meaningful statistical pooling and could have produced biased summary estimates with limited clinical interpretability. Narrative synthesis was therefore considered the most appropriate approach.

### 2.2. Literature Search Strategy

We systematically searched PubMed/MEDLINE, Embase, Scopus, Web of Science Core Collection, and the Cochrane Library for studies evaluating metformin in the context of fertility-sparing treatment for atypical endometrial hyperplasia (AEH), endometrial intraepithelial neoplasia (EIN), and endometrioid endometrial cancer (EEC). The search covered the period from database inception through 31 May 2026.

The search concepts concerned metformin, atypical endometrial hyperplasia/endometrial intraepithelial neoplasia, endometrial cancer, and fertility-sparing or conservative treatment.

The original executable database-specific search strings and individual database retrieval counts were unavailable for verification. Consequently, the Boolean operators, controlled vocabulary, field tags, and database-specific adaptations used in the original searches could not be retrospectively confirmed. Supplementary Table S2 provides an illustrative conceptual framework only; it is not a historical search log or a record of verified executable queries. The available documentation does not permit an explanation of the relatively low total retrieval count of 87 records.

To reduce the chance of missing relevant evidence, the reference lists of all eligible studies and relevant review articles were manually searched for additional publications not identified by the electronic database search. Only studies published in English were considered eligible for inclusion. The number of non-English records identified or excluded was not documented separately; therefore, the effect of the language restriction on the search yield could not be quantified. The literature search, study selection and reporting were guided by the Preferred Reporting Items for Systematic Reviews and Meta-Analyses (PRISMA) 2020 statement.

### 2.3. Study Selection

Following removal of duplicate records, two reviewers independently screened the titles and abstracts of all retrieved studies according to the predefined eligibility criteria. Studies considered potentially eligible by either reviewer proceeded to full-text assessment. The same two reviewers independently evaluated the full-text articles to determine final eligibility for inclusion in the systematic review.

Any disagreements regarding study selection were resolved through discussion and consensus. When consensus could not be reached, a third reviewer was consulted to adjudicate the final decision. Reasons for exclusion of studies at the full-text stage were documented and are presented in the PRISMA 2020 flow diagram (Figure 1).

The study selection process was conducted in accordance with the PRISMA 2020 Statement to ensure a transparent and systematic description to the identification and selection of eligible studies.

### 2.4. Eligibility Criteria

Eligibility criteria were formulated according to the population, intervention, comparator, and outcome (PICO) framework. Studies were eligible if they included women of reproductive age with atypical endometrial hyperplasia (AEH), endometrial intraepithelial neoplasia (EIN), and/or histologically confirmed endometrioid endometrial cancer (EEC), typically grade 1 and stage IA, who were managed with fertility-sparing intent. Studies of mixed populations were eligible when they included patients with AEH/EIN and/or early-stage EEC relevant to the review question. Diagnosis-specific data were extracted separately when available. The intervention of interest was metformin administered as part of a fertility-sparing strategy, including metformin monotherapy; metformin combined with oral progestins (e.g., medroxyprogesterone acetate or megestrol acetate); metformin combined with an LNG-IUS; metformin combined with hysteroscopic resection and hormonal therapy; or another conservative regimen incorporating metformin. Studies were eligible regardless of comparator status. The primary outcomes comprised oncologic, reproductive, and safety outcomes. Eligible designs included randomized controlled trials, prospective studies, retrospective cohort studies, and other observational studies evaluating metformin-containing fertility-sparing strategies in women with AEH/EIN or early-stage EEC.

Review articles, clinical guidelines, conference abstracts, editorials, letters to the editor, animal studies, and study protocols without reported clinical outcomes were excluded. Only articles published in English were eligible. Additional exclusion criteria concerned irrelevant populations, interventions, or outcomes. Duplicate publications and studies involving only experimental animal models without clinical translation were also excluded.

### 2.5. Data Extraction

After eligible studies had been selected, data on the prespecified variables of interest were extracted using a standardized form developed a priori to ensure consistent and complete data collection. Two reviewers independently extracted data from each study. The extracted information included study, patient, disease, and treatment characteristics, as well as outcomes, as summarized in Table 1. For studies that included mixed populations of patients with AEH/EIN and early-stage EEC, subgroup-specific data were extracted separately when reported. When outcomes were available only for the combined cohort, pooled data were retained and synthesized narratively. Subgroup-specific outcomes were not derived when they were unavailable in the original publications. The primary outcomes included recurrence, disease progression, time to complete response (CR), pregnancy, live birth, and adverse events. Because this systematic review analyzed previously published literature, ethics approval and informed consent were not required. Multiple publications derived from the same underlying patient cohort were treated as separate reports but not as independent cohorts when calculating the total number of unique patients. Clinical events reported in overlapping publications, including recurrences and live births, were not summed across reports. Because the findings were synthesized narratively and no pooled event counts or outcome rates were calculated, outcomes were retained at the individual-report level and interpreted according to the specific research question addressed by each publication. When the same patients or potentially overlapping events were represented in more than one report, the publications were considered complementary analyses of a single cohort rather than independent evidence.

### 2.6. Risk of Bias Assessment

The methodological quality of the included studies was assessed according to study design. Because most were observational cohort studies, the Newcastle–Ottawa Scale (NOS) was used to evaluate study selection, comparability between groups, and outcome assessment. Randomized controlled trials were evaluated using the Cochrane Risk of Bias 2 (RoB 2) tool, which assesses bias arising from the randomization process, deviations from intended interventions, missing outcome data, outcome measurement, and selection of reported results.

The quality assessment was performed independently by two reviewers. Any disagreements were resolved through discussion, and when necessary, a third reviewer was consulted to reach consensus.

A formal certainty-of-evidence assessment using the GRADE framework was not performed because the review aimed to provide a narrative synthesis of a limited and clinically heterogeneous body of evidence. Consequently, the overall strength of the evidence discussed in this review reflects the authors’ interpretation based on the methodological quality, risk of bias, consistency of findings, and clinical heterogeneity of the included studies, rather than a standardized certainty-of-evidence assessment.

### 2.7. Data Synthesis

Given the substantial heterogeneity among the included studies, a quantitative meta-analysis was not considered appropriate. Heterogeneity was present at multiple levels. Clinical heterogeneity arose from differences in patient populations (AEH/EIN versus early-stage EEC), fertility-sparing treatment strategies, metformin dosages, treatment duration, and follow-up protocols. Methodological heterogeneity resulted from the inclusion of randomized controlled trials, prospective studies and retrospective cohort studies with different study designs and methodological quality. Differences in the timing of response assessment, biopsy and surveillance protocols, definitions and reporting of oncologic and reproductive outcomes, and incomplete reporting of a number of clinically relevant endpoints accounted for the heterogeneity of outcomes. Although complete response (CR) was consistently defined as histological regression or the absence of residual AEH/EIN or EEC, the timing of assessment and reporting methods varied across studies. Collectively, these sources of heterogeneity precluded meaningful statistical pooling and could have resulted in misleading summary estimates. Therefore, a narrative synthesis was considered the most appropriate methodological approach.

## 3. Results

The initial systematic literature search identified 87 records. After removal of 52 duplicates, 35 records underwent title and abstract screening. Twelve records were excluded at this stage because they did not meet the predefined eligibility criteria based on population, intervention, study design, or relevance to fertility-sparing treatment with metformin. The remaining 23 articles underwent full-text assessment. Of these, 15 were excluded because they did not meet the predefined inclusion criteria, leaving eight eligible publications for inclusion in the systematic review. The study selection process is summarized in the PRISMA 2020 flow diagram (Figure 1).

### 3.1. Study Characteristics

The eight included publications represented approximately 795 unique patients with atypical endometrial hyperplasia/endometrial intraepithelial neoplasia or early-stage endometrioid endometrial cancer, after accounting for overlap between two reports derived from the same underlying patient cohort. Reports by Wang et al. [24] and Liu et al. [26] were identified as based on the same underlying cohort at the Obstetrics and Gynecology Hospital of Fudan University during the same recruitment period (January 2017 to August 2019). The two studies had different research questions and slightly different analytic samples (285 and 286 patients, respectively) but were not considered independent cohorts in the calculation of the total number of unique patients.

Study designs included randomized controlled trials, prospective studies, and retrospective cohort studies. Characteristics of the included studies are summarized in Table 1.

### 3.2. Oncologic and Reproductive Outcomes

Oncologic outcomes varied across populations, treatment groups, and assessment times. Mitsuhashi et al. reported complete response in 61/63 patients (97%) [21]. Acosta-Torres et al. reported complete response in 63/92 patients overall (69%), including 23/34 (68%) receiving progestin plus metformin and 40/58 (69%) receiving progestin alone [22]. Reported recurrence estimates included 8/61 (13.1%) in Mitsuhashi et al. and 26/76 (34.2%) in Jing et al. [21,25]. Live-birth outcomes are reported by study and denominator below, rather than as a single range. Table 2 summarizes the reported outcomes.

### 3.3. Oncologic Outcomes

All included studies reported generally favorable oncologic outcomes with metformin-containing fertility-sparing strategies. The study-specific complete response estimates described above require cautious interpretation because study design, patient populations, treatment regimens, and assessment times differed. Some studies suggested that adding metformin to progestin therapy may improve outcomes, particularly among women with atypical endometrial hyperplasia. Yang et al. [23] reported improved early complete response rates with megestrol acetate plus metformin, whereas Gu et al. [28] reported promising results with LNG-IUS plus metformin. In contrast, Acosta-Torres et al. [22] did not demonstrate a clear benefit associated with metformin. Overall, the available evidence suggests that metformin may be beneficial as an adjunct to fertility-sparing treatment, although further prospective studies are needed to establish its clinical value.

### 3.4. Recurrence Outcomes

Disease recurrence remained an important concern across the included studies, even among women who initially achieved complete remission. Reported recurrence rates varied widely, from 13.1% to 34.2%. Several studies suggested that obesity, insulin resistance, and polycystic ovary syndrome may be associated with a higher risk of recurrence and poorer long-term outcomes. Metformin-containing regimens generally had acceptable recurrence profiles, but the available data do not eliminate the need for rigorous long-term follow-up. These findings underscore the importance of continued surveillance after treatment completion, particularly among patients with persistent metabolic risk factors.

Wang et al. [27] reported recurrence in 5/26 patients (19.2%) receiving combination treatment in Section 3, whereas the Abstract and Discussion reported 22.7%; this unresolved discrepancy limits interpretation of the study-specific recurrence estimate.

### 3.5. Reproductive Outcomes

Live-birth outcomes were reported using different denominators, limiting direct comparisons. Mitsuhashi et al. reported live births in 14/31 women wishing to conceive (45%) [21]. Acosta-Torres et al. reported live births in 16/92 women overall (17%): 2/34 (6%) receiving progestin plus metformin and 14/58 (24%) receiving progestin alone. The number attempting conception could not be determined, so the denominators included all women in each treatment group [22]. Jing et al. reported live births in 29/79 patients overall (36.7%), and separately in 27/55 nulliparous women wishing to conceive after complete response (49.1%) [25]. These proportions should not be interpreted as directly comparable estimates of reproductive efficacy. Assisted reproductive technologies were frequently used [21,22,25].

### 3.6. Adverse Events and Treatment Adherence

Reporting of adverse events and treatment adherence was heterogeneous across the included studies. Six of the eight studies provided at least some safety or adherence information, whereas Acosta-Torres et al. [22] and Wang et al. [24] did not report adverse-event, toxicity-related discontinuation, or dose-reduction outcomes. Mitsuhashi et al. [21] provided the most detailed metformin-specific data. No severe toxicity was observed; grade 1 and grade 2 diarrhea occurred in six and three patients, respectively, and grade 1 and grade 2 nausea occurred in six and four patients, respectively. Gastrointestinal symptoms occurred mainly at a metformin dose of 2250 mg/day and generally resolved after dose reduction to 1500 mg/day. Metformin was temporarily interrupted in two patients and permanently discontinued in two patients because of grade 2 nausea. In the randomized study by Yang et al. [23], grade 1–2 diarrhea was more frequent with metformin plus megestrol acetate than with megestrol acetate alone (15.8% vs. 4.1%), but no fatal adverse events were reported. Wang et al. [27] reported mild, tolerable nausea in three patients receiving the combined regimen, without treatment interruption, and no severe adverse effects. Gu et al. [28] reported fewer adverse reactions with LNG-IUS plus metformin than with oral progestin plus metformin (12.8% vs. 34.6%); because both groups received metformin, these regimen-level findings cannot be attributed specifically to metformin. Jing et al. [25] reported no grade 3–4 adverse events related to metformin or megestrol acetate, while Liu et al. [26] reported no serious drug-related adverse events and no patients with poor medication adherence. Overall, metformin was generally tolerated, but inconsistent reporting precluded estimation of a pooled discontinuation or dose-reduction rate.

### 3.7. Quality Assessment of Included Studies

The results of the methodological quality assessment are summarized in Table 3 and Table 4. Overall, the observational studies were judged to be of moderate-to-high methodological quality. The main methodological limitations included the retrospective design of most studies, limited comparability between treatment groups, and the absence of concurrent control groups. Two studies were considered high quality, whereas the remaining studies were classified as moderate quality. Both randomized controlled trials were judged as presenting some concerns regarding the overall risk of bias.

**Table 3:** Methodological quality assessment of observational studies using the Newcastle–Ottawa Scale domains.

Study	Selection	Comparability	Outcome Assessment	Overall Quality
Mitsuhashi et al., 2019 [21]	Adequate	Limited	Adequate	Moderate
Acosta-Torres et al., 2020 [22]	Adequate	Moderate	Adequate	Moderate
Wang et al., 2021 [24] * (PCOS subgroup)	Adequate	Adequate	Adequate	High
Liu et al., 2022 [26] *	Adequate	Adequate	Adequate	High
Jing et al., 2022 [25]	Adequate	Limited	Adequate	Moderate
Gu et al., 2026 [28]	Adequate	Moderate	Adequate	Moderate

* Wang et al. (2021) [24] and Liu et al. (2022) [26] were derived from the same underlying Fudan University cohort and should not be considered independent patient populations. The slight difference in sample size reflects study-specific eligibility criteria.

**Table 4:** Risk of Bias Assessment of Randomized Controlled Trials Using the Cochrane Risk of Bias 2 (RoB2) Tool.

Domain	Yang et al., 2020 [23]	Wang et al., 2024 [24]
Bias arising from the randomization process	Low risk	Some concerns
Bias due to deviations from intended interventions	Some concerns	Some concerns
Bias due to missing outcome data	Some concerns	Some concerns
Bias in measurement of the outcome	Low risk	Some concerns
Bias in selection of the reported result	Some concerns	Some concerns
Overall risk of bias	Some concerns	Some concerns

Note. The outcome-measurement judgments refer to histological complete response. Yang et al. reported blinded pathological assessment. For Wang et al., histological assessment and a common three-month surveillance schedule were described, but assessor blinding was not reported. Some concerns were therefore assigned because assessment involves interpretation, although the report did not establish that knowledge of treatment influenced the findings. This domain judgment should not automatically be extrapolated to recurrence or reproductive outcomes.

### 3.8. Interpretation

High quality: low-to-moderate risk of bias with appropriate patient selection and adjustment for confounding factors.

Moderate quality: acceptable methodology, but limited by retrospective design, lack of optimal comparability, or absence of a concurrent control group.

## 4. Discussion

The objective of this systematic review was to assess the role of metformin in the fertility-sparing treatment of atypical endometrial hyperplasia and early-stage endometrioid endometrial cancer, with particular emphasis on oncologic and reproductive outcomes. Metformin-containing regimens were associated with high rates of histological regression in AEH and early-stage EEC, suggesting a potential additive or synergistic effect when metformin is combined with progestin therapy or other standardized interventions [21,28]. Recurrence after complete response underscores the need for continued surveillance [21,25]. Reproductive outcomes varied substantially, and differences in denominators and attempts at conception preclude direct comparisons [21,22,25]. Gu et al. reported higher complete response, pregnancy, and live birth rates and lower recurrence with LNG-IUS plus metformin than with megestrol acetate plus metformin; both groups received metformin, so this comparison does not isolate its contribution [28]. Higher BMI, older age, and cancer histology were associated with poorer outcomes in the analyses reported by Gu et al. [28], while Acosta-Torres et al. identified cancer histology as a predictor of reduced treatment response [22]. The included reports differed in treatment structure, metabolic characteristics, and outcome reporting. This clinical heterogeneity is important when interpreting the findings. Women with AEH/EIN and those with early-stage EEC differ in biological behavior, baseline progression risk, and expected response to fertility-sparing treatment. Fertility-sparing strategies also varied considerably and included oral progestins, LNG-IUS-based regimens, hysteroscopic approaches, and different metformin doses and treatment durations. Follow-up periods and outcome definitions varied across studies. Consequently, the observed clinical outcomes cannot be attributed solely to metformin, and direct comparisons must be interpreted cautiously. This heterogeneity also limits the ability to identify the optimal regimen, metformin dose, or patient subgroup most likely to benefit [21,22,25,26,28]. Metformin doses ranged from 750 to 2250 mg/day in studies that reported dosing, whereas several studies provided no detailed dose information. Although this variation may have contributed to differences in complete response rates, the available data do not demonstrate a clear dose–response relationship. High complete response rates were reported with both titrated and fixed-dose regimens, whereas other studies using comparable doses found no clear additional effect on remission. The independent effect of metformin dose cannot be distinguished from differences in diagnosis, concomitant hormonal therapy, treatment duration, patient metabolic characteristics, and the timing of response assessment. Prospective studies using standardized dosing protocols are needed to determine whether metformin dose independently influences treatment response. The findings should therefore be interpreted cautiously and should not be considered definitive evidence of treatment efficacy.

Although most included studies were judged to be of moderate-to-high methodological quality according to the Newcastle–Ottawa Scale and the Cochrane Risk of Bias 2 tool, no formal certainty-of-evidence assessment (e.g., GRADE) was performed. Conclusions regarding the overall strength of the evidence therefore represent the authors’ narrative interpretation and should be considered cautiously. Most available data were derived from retrospective single-center studies, and some studies lacked concurrent control groups or had limited comparability between treatment groups.

The methodological limitations of the randomized studies require differentiated interpretation. Yang et al. [23] used an open-label design, with patients and treating physicians aware of allocation, but treatment assignment was concealed before enrolment and the assessing pathologists were blinded. Thus, knowledge of treatment allocation could potentially influence treatment-related behaviour, whereas blinded histological assessment reduced concerns about subjective outcome measurement. In Wang et al. [27], blinding and allocation concealment were not explicitly reported; these reporting gaps should not be interpreted as evidence that these safeguards were absent. Nevertheless, because histological response assessment involves interpretation and assessor blinding was not established, the outcome-measurement domain for Wang et al. was judged as having some concerns.

Both trials scheduled hysteroscopic assessments approximately every three months, without a reported difference in planned assessment intervals between treatment arms. However, Yang et al. [23] documented delayed or cancelled examinations and analysed response within 16 and 32 weeks rather than at the originally planned three- and six-month time points. Only 125 and 136 of the 150 randomized participants, respectively, contributed to these analyses; the 16-week analysis included 58/74 control-group participants and 67/76 combination-group participants. Consequently, early response estimates may be influenced by assessment timing and incomplete outcome ascertainment, rather than reflecting treatment efficacy alone. In Wang et al. [27], two control-group participants crossed over to combination treatment after six months and were included in the combination group in the nine-month analysis, limiting interpretation according to the original randomized allocation.

Recurrence comparisons also require caution because of losses to follow-up and differences in follow-up duration, particularly between studies. Yang et al. [23] reported a median post-remission follow-up of 33.4 months and nine patients lost to follow-up (three in the combination group and six in the control group). Wang et al. [27] reported mean follow-up durations of 13.99 and 13.08 months in the combination and control groups, respectively, and three patients lost after treatment without specifying their allocation in that passage. The timing of surveillance determines when remission or recurrence is documented, not necessarily when it biologically occurs. These limitations reduce confidence in the reported treatment effects without establishing the direction or magnitude of bias.

Experimental and translational studies provide insight into mechanisms through which metformin may improve the efficacy of fertility-sparing treatment in AEH and EEC. First, metformin may target metabolic, endocrine, and oncogenic dysregulation, which is particularly relevant in patients with obesity or insulin resistance, by acting as a systemic disease-modifying agent rather than a purely cytotoxic drug [29,30]. Second, activation of AMP-activated protein kinase (AMPK) inhibits mTORC1 signaling, S6 kinase (S6K1), and EIF4E-mediated translation initiation, thereby reducing protein synthesis and ribosomal biogenesis [31]. This pathway promotes cell-cycle arrest and apoptotic sensitivity, suppressing endometrial epithelial proliferation. These effects may be particularly relevant in AEH and well-differentiated EEC, in which mTOR signaling is frequently upregulated [32,33]. Metformin may also attenuate PI3K/AKT/mTOR signaling and endometrial oncogenesis through indirect AMPK-mediated inhibition of mTORC1, reduced upstream insulin/IGF-1 signaling, and decreased AKT phosphorylation in certain cellular contexts [34,35,36]. These mechanisms are relevant because fertility-sparing therapy relies on the reversibility of hyperplastic epithelium, which depends partly on suppression of proliferative signaling. Metformin may also mitigate progesterone resistance through upregulation of progesterone receptor expression, downregulation of estrogen receptor-α signaling, and restoration of epithelial responsiveness to progestins [37,38]. Potential functional consequences include enhanced decidualization in endometrial stromal cells, increased sensitivity to medroxyprogesterone acetate and LNG-IUS therapy, and improved histological regression with combination therapy [39]. These mechanistic findings provide biological plausibility for using metformin in fertility-sparing management but do not establish clinical efficacy. Figure 2 summarizes the proposed mechanisms through which metformin may influence oncologic and reproductive outcomes in AEH/EIN and early-stage EEC.

### 4.1. Strengths and Clinical Implications

A notable strength of this systematic review is its focus on the emerging role of metformin within fertility-preserving treatment strategies for atypical endometrial hyperplasia and early-stage endometrioid endometrial cancer. By bringing together evidence from studies evaluating different metformin-containing treatment approaches, the review provides an overview of both oncologic and reproductive outcomes in a patient population for whom fertility preservation remains a major clinical concern.

From a clinical perspective, the available evidence suggests a potential adjunctive role for metformin in combination with conventional progestin-based therapy. The benefits may be particularly relevant among women with obesity, insulin resistance, polycystic ovary syndrome, or other metabolic abnormalities, in whom endocrine and metabolic factors may contribute to disease development or treatment resistance. Although the evidence does not support the routine use of metformin in all patients undergoing fertility-sparing treatment, the observed treatment responses and biological rationale warrant further investigation in well-designed prospective studies.

Our findings should also be considered in the context of previous systematic reviews. Chae-Kim et al. found that adding metformin to progestin therapy was associated with a lower recurrence rate but did not significantly improve complete response, pregnancy, or live birth rates [40]. More recently, a 2025 Cochrane review evaluated a broad range of fertility-sparing strategies for women with AEH/EIN and early-stage EEC, including metformin-containing regimens, and concluded that the evidence remained limited because of the small number of randomized studies and the substantial clinical heterogeneity [41]. Our findings are consistent with these conclusions. However, unlike the previous reviews, the present review focuses specifically on metformin as an adjunct to fertility-sparing treatment, includes more recently published clinical studies, and provides a detailed narrative appraisal of the clinical and methodological heterogeneity that limits interpretation of the evidence.

### 4.2. Limitations

Several limitations should be considered when interpreting this systematic review. Most included studies were retrospective, increasing the risk of selection bias and limiting adjustment for potential confounders. Patient characteristics, treatment protocols, metformin dosage, treatment duration, and assessed outcomes varied considerably. Direct comparisons were further hindered by inconsistent reporting of reproductive outcomes, including pregnancy and live birth rates. The small sample sizes of several studies may also limit the generalizability of their findings. In addition, two publications were derived from the same underlying patient cohort but addressed different research questions. This overlap was considered when calculating the total number of unique patients and interpreting the evidence. These limitations underscore the need for larger, well-designed prospective studies to define the role of metformin in fertility-sparing treatment for AEH/EIN and early-stage EEC.

The complete database-specific search histories and individual database retrieval counts were not retained, limiting the reproducibility and retrospective verification of the literature search. In addition, only English-language publications were eligible, and the number of potentially relevant non-English records was not documented separately. Therefore, language bias cannot be excluded, and the contribution of the language restriction to the relatively small evidence base could not be determined.

A further limitation was the lack of a formal certainty-of-evidence framework (e.g., GRADE). Accordingly, statements regarding the overall strength of the available evidence should be interpreted as the authors’ narrative assessment rather than a standardized certainty-of-evidence evaluation. In addition, publication bias was not formally assessed because the limited number of included studies and their substantial clinical and methodological heterogeneity precluded meaningful evaluation using conventional methods, such as funnel plots. Consequently, the possibility of publication bias cannot be excluded.

Despite the encouraging findings, several critical questions remain regarding the optimal integration of metformin into fertility-sparing strategies for AEH/EIN and early-stage EEC. Future research should refine patient selection, optimize treatment protocols, and generate high-quality prospective evidence on the oncologic and reproductive effects of metformin-containing regimens. The predominance of retrospective studies and the small number of randomized trials limit the strength of the available evidence despite the generally moderate-to-high methodological quality of the included studies.

### 4.3. Research Gaps and Future Perspectives

Although evidence supports the use of metformin as an adjunct to fertility-sparing treatment in women with AEH/EIN and early-stage EEC, several important uncertainties remain. One major limitation is the considerable variation in metformin dosing regimens across studies, which hinders comparisons and prevents determination of the optimal therapeutic dose. Future randomized controlled trials should establish the most effective and best-tolerated dosing regimen.

Another unresolved question concerns the optimal duration of metformin treatment and whether therapy should continue after complete remission. Long-term prospective studies are needed to establish evidence-based treatment and maintenance strategies. Patient selection is equally important. Women with obesity, insulin resistance, or polyendocrine metabolic ovarian syndrome may be particularly likely to receive metformin, but the populations most likely to benefit remain insufficiently defined. Predictive models integrating clinical, metabolic, and molecular characteristics may facilitate more individualized treatment decisions.

An additional evidence gap concerns molecular classification. Contemporary endometrial cancer frameworks distinguish POLE-mutated (POLEmut), mismatch repair-deficient (MMRd), no specific molecular profile (NSMP), and p53-abnormal (p53abn) tumors. However, the predominantly retrospective cohorts included in this review did not systematically stratify patients according to these molecular subtypes. Therefore, it remains uncertain whether molecular subtype influences complete response, recurrence, or reproductive outcomes following metformin-containing fertility-sparing treatment. Although MMR deficiency and p53 abnormalities may identify biologically higher-risk disease, the prognostic and predictive value of molecular classification in the fertility-sparing setting remains insufficiently validated, and molecular subtype should not yet be used as a stand-alone selection criterion [2,42]. Future prospective studies should integrate standardized molecular profiling with clinical and metabolic risk assessment, including BMI, insulin resistance, polycystic ovary syndrome, diabetes, and metabolic syndrome. Combined molecular–metabolic models may enable more precise candidate selection, individualized counselling, treatment stratification, and surveillance while helping to identify patients most likely to benefit from adjunctive metformin.

Another important research objective is the identification of reliable biomarkers. No validated biomarkers currently predict treatment response, relapse risk, or reproductive outcomes. Future studies should evaluate progesterone receptor expression, metabolic parameters, genetic alterations, and other molecular markers that may guide therapeutic decision-making. Emerging liquid-biopsy approaches, including circulating microRNAs (miRNAs), may provide minimally invasive tools for patient stratification and have potential applications in longitudinal monitoring of treatment response [43,44]. Although circulating biomarkers have demonstrated potential diagnostic and prognostic value in endometrial cancer, their ability to specifically predict the response to metformin in women with AEH/EIN or early-stage EEC remains unknown and requires prospective validation [43,44].

Women with polyendocrine metabolic ovarian syndrome (PMOS; formerly polycystic ovary syndrome [PCOS]) [45] represent an important subgroup for future research. Studies using the historical PCOS terminology reported associations with poorer fertility-sparing treatment outcomes [24,26]. Further prospective studies are needed to determine whether adjunctive metformin improves oncologic and reproductive outcomes in this population.

Emerging evidence also suggests that combining an LNG-IUS with metformin may produce better oncologic and reproductive outcomes than conventional progestin-based regimens. However, most existing studies are retrospective and provide limited information on long-term benefits. Well-designed multicenter randomized controlled trials are needed to confirm these preliminary findings and establish the role of LNG-IUS plus metformin in clinical practice.

Another weakness of the current literature is the inconsistent reporting of reproductive outcomes. Although fertility preservation is the principal aim of conservative treatment, studies report pregnancy rates, live birth rates, and assisted reproductive technology outcomes inconsistently. Future prospective studies and registries should adopt standardized reporting. At a minimum, reports should include the number of women attempting conception, time to pregnancy, mode of conception (spontaneous or assisted), type of assisted reproductive technology used, pregnancy and miscarriage rates, and cumulative live birth rates. Outcomes should also be reported in relation to completion of fertility-sparing treatment and follow-up duration to permit meaningful comparisons across studies. Long-term oncologic outcomes are also poorly characterized. Most studies had short follow-up periods, limiting evaluation of recurrence and long-term disease control. Future registries and long-term follow-up studies are needed to characterize recurrence risk and survival outcomes more accurately.

Finally, the molecular mechanisms through which metformin may increase progesterone sensitivity and improve treatment response require further investigation. Experimental studies implicate AMPK activation, PI3K/AKT/mTOR signaling, insulin-mediated pathways, and progesterone receptor modulation, but the interactions among these mechanisms remain incompletely understood.

Future investigations should include large prospective multicenter studies with standardized treatment protocols, detailed reporting of reproductive outcomes, and integration of molecular and metabolic biomarkers. Such studies will help define the precise role of metformin in fertility-sparing treatment and support the development of individualized therapeutic strategies. Published trial protocols also describe prospective evaluation of metformin-containing regimens, but provide no completed clinical outcome data and were not included as outcome studies [46,47].

## 5. Conclusions

Metformin has emerged as a promising adjunct to fertility-sparing management for AEH/EIN and early-stage EEC. Although metformin-containing regimens were generally associated with favorable oncologic and reproductive outcomes, the substantial heterogeneity of the available evidence precludes firm conclusions regarding the magnitude of benefit or the optimal therapeutic strategy. The potential effects of metformin are biologically plausible because the drug influences insulin resistance, metabolic dysfunction, and proliferative signaling pathways involved in endometrial carcinogenesis. These mechanisms may also enhance progesterone responsiveness, which is critical to fertility-sparing treatment. Nevertheless, the current evidence remains insufficient. Most available studies are retrospective, include relatively small patient populations, and differ substantially in treatment protocols, follow-up duration, and outcome reporting. Consequently, the clinical benefit attributable specifically to metformin remains uncertain. Well-designed prospective randomized studies are needed to define its optimal role, identify patients most likely to benefit, and establish standardized treatment strategies. At present, metformin appears to be a promising component of individualized fertility-sparing management, particularly for women with obesity, insulin resistance, or other metabolic risk factors; however, its routine incorporation into clinical practice cannot yet be universally recommended.

## Figures and Tables

**Figure 1:**
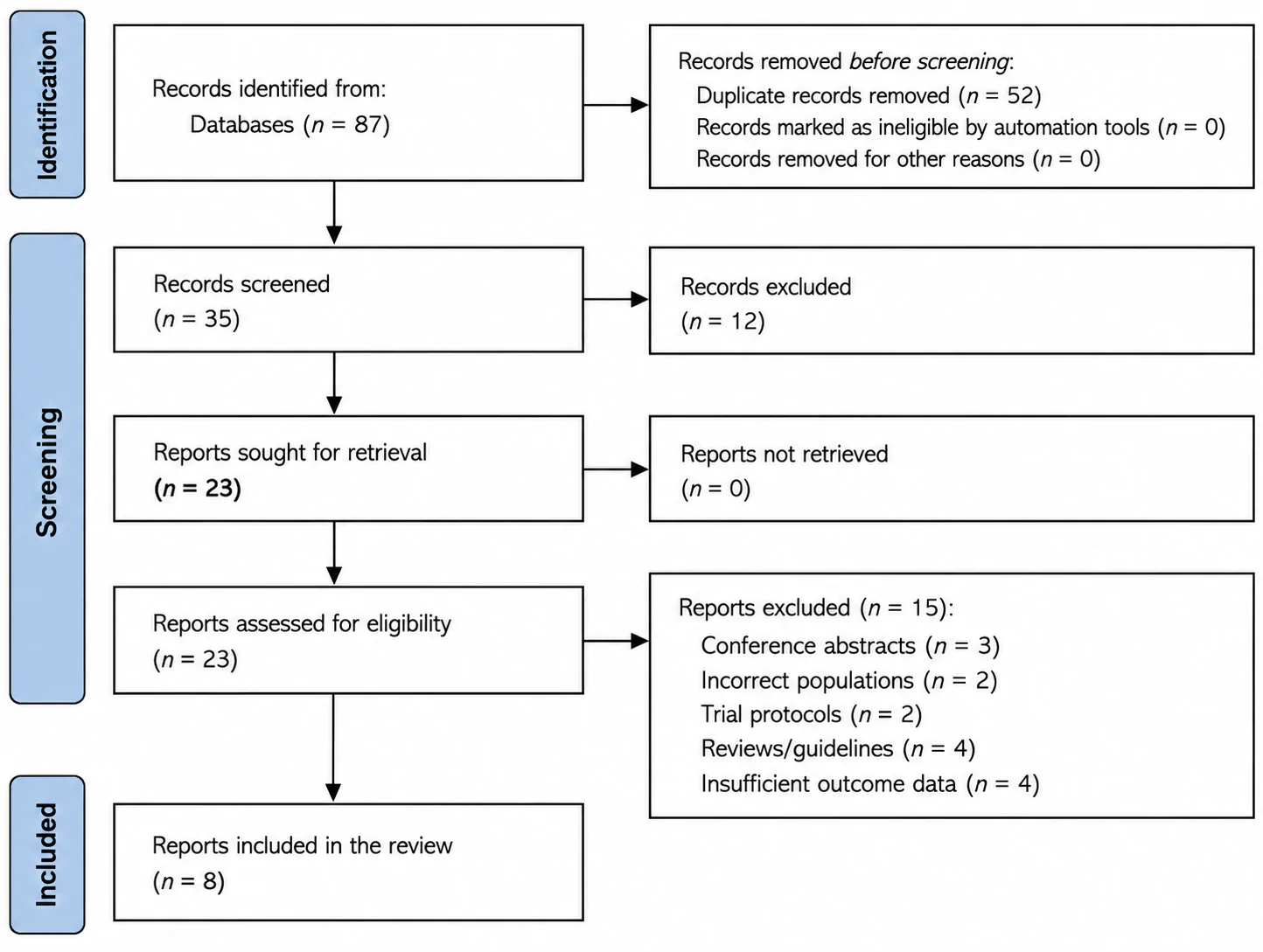
PRISMA 2020 Flow Diagram.

**Figure 2:**
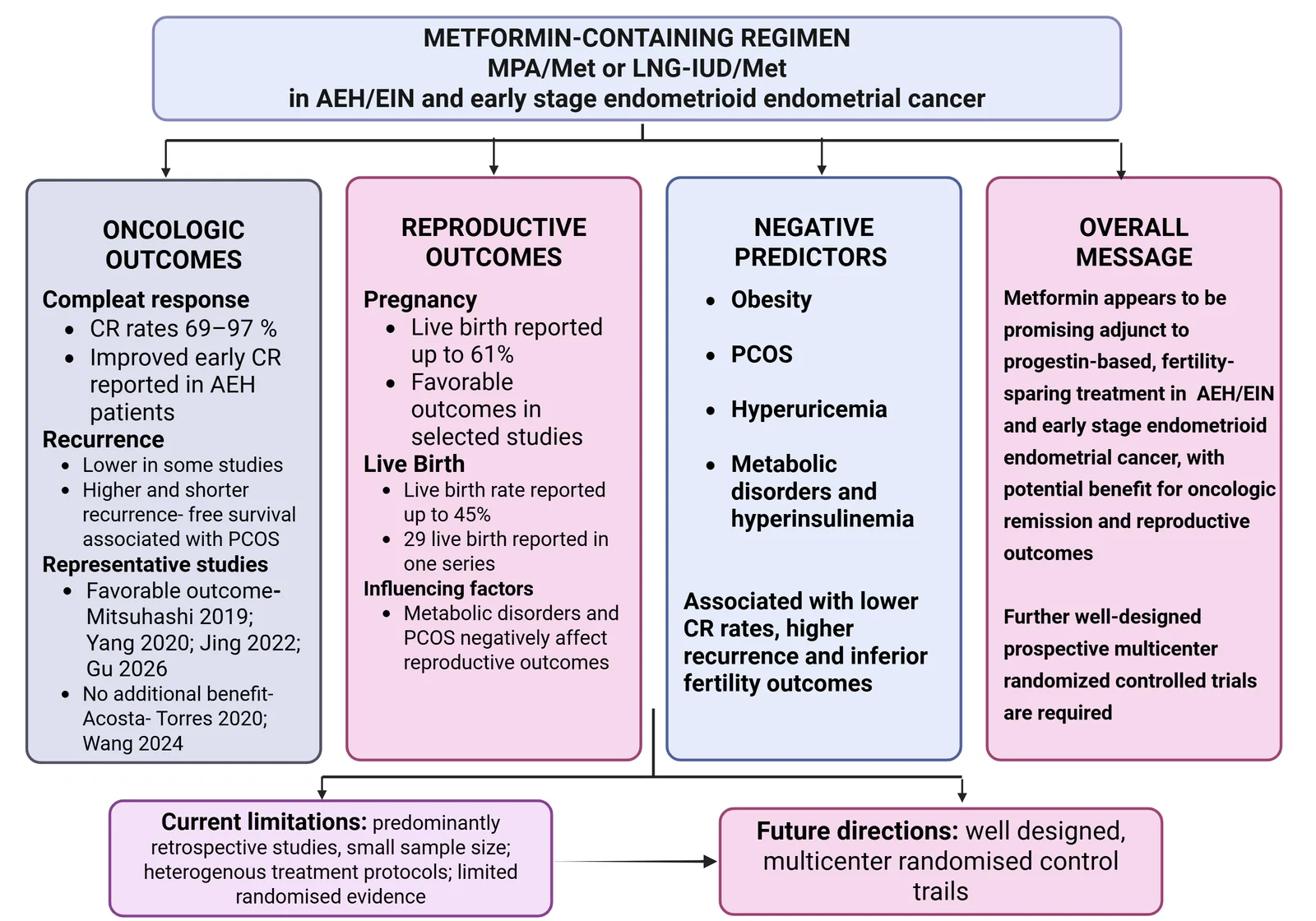
Schematic overview of the available evidence regarding metformin-containing fertility-sparing treatment strategies in women with atypical endometrial hyperplasia/endometrial intraepithelial neoplasia (AEH/EIN) and early-stage endometrioid endometrial cancer. The figure summarizes the main oncologic and reproductive outcomes, negative predictors of response, current limitations of the available evidence, and future research directions [21,22,23,24,25,28]. Created in https://BioRender.com.

**Table 1:** Characteristics of Included Studies Evaluating Metformin-Containing Fertility-Preserving Treatment Strategies in Women with AEH and EEC.

Study	Design	N	Diagnosis	Age	BMI	Treatment/Comparator	Metformin Dose	Follow-Up
Mitsuhashi 2019 [21]	Retrospective cohort	63	AEH 21/EC 42	35, median range 26–44	30.9, median range 15.4–50.8	MPA + metformin	750–2250 mg/day	57 months
Acosta-Torres 2020 [22]	Retrospective cohort	92	AH/EIN 54/EC 38	35, median	37.7, median	Progestin ± metformin	NR; metformin duration 18.3 months	28.4 months
Yang 2020 [23]	RCT	150	AEH 102/EEC 48	NR	NR	MA vs. MA + metformin	500 mg three times daily	30 weeks; post-CR follow-up 33.4 months
Wang 2021 [24]	Retrospective cohort	285	AEH 209/EEC 76	32, median range 20–45	24.8, median range 16.7–51.6	Progestin-based therapy; PCOS vs. non-PCOS	500 mg TID when used	22.8 months
Jing 2022 [25]	Retrospective cohort	79	AEH 31/EEC 48	30, median IQR 27–33	23.4, median IQR 20.0–27.5	Hysteroscopic curettage + MA + metformin	1500 mg/day	39.5 months
Liu 2022 [26]	Retrospective cohort	286	EAH 209/EEC 77	18–45	NR	Progestin-based therapy; metabolic subgroup analysis	NR	NR
Wang 2024 [27]	Prospective randomized study	60	AEH/G1 EAC	20–42	NR	MA vs. MA + metformin	850 mg twice daily	12 months
Gu 2026 [28]	Retrospective cohort	65	AEH 51/EC 14	≤45	NR	LNG-IUS + metformin vs. MA + metformin	NR	NR

Abbreviations: AEH, atypical endometrial hyperplasia; EAH, endometrial atypical hyperplasia; AH, atypical hyperplasia; EIN, endometrial intraepithelial neoplasia; EC, endometrial cancer; EEC, endometrioid endometrial cancer; EAC, endometrial adenocarcinoma; G1, grade 1; RCT, randomized controlled trial; BMI, body mass index; PCOS, polycystic ovary syndrome; MA, megestrol acetate; MPA, medroxyprogesterone acetate; LNG-IUS, levonorgestrel-releasing intrauterine system; CR, complete response; IQR, interquartile range; TID, three times daily; NR, not reported. The original diagnostic terminology used by the individual studies (e.g., AEH, EAH, or AH) was retained in the table to accurately reflect the terminology reported by the study authors.

**Table 2:** Oncologic and Reproductive Outcomes of Metformin-Containing Fertility-Preserving Treatment Strategies.

Study	Complete Response	Recurrence	Reproductive Outcomes	Principal Findings
Mitsuhashi et al., 2019 [21]	61/63 (97%)	8/61 (13.1%)	Pregnancy: 19/31 (61%); live birth: 14/31 (45%); women wishing to conceive	Excellent long-term oncologic and reproductive outcomes with MPA plus metformin
Acosta-Torres et al., 2020 [22]	Overall: 63/92 (69%); Prog-Met: 23/34 (68%); Prog: 40/58 (69%)	Overall: 14/63 (22%); Prog-Met: 4/23 (17%); Prog: 10/40 (25%)	Live birth: Prog-Met 2/34 (6%); Prog 14/58 (24%); overall 16/92 (17%). All women per group; attempts at conception unknown	Addition of metformin did not significantly improve response
Yang et al., 2020 [23]	Improved early response	NR	NR	Enhanced 16-week CR, particularly in AEH
Wang et al., 2021 [24]	Lower CR in women with PCOS	Shorter recurrence-free survival	Inferior reproductive outcomes	PCOS negatively influenced treatment outcomes
Jing et al., 2022 [25]	96.2%	26/76 (34.2%)	Live birth: 29/79 (36.7%) overall; 27/55 (49.1%) in nulliparous women wishing to conceive after CR	Combined hysteroscopy, MA and metformin achieved high remission rates
Liu et al., 2022 [26]	Reduced CR in obese and metabolically compromised patients	NR	NR	Obesity, PCOS and hyperuricemia adversely affected response
Wang et al., 2024 [27]	Similar between groups	Similar between groups	NR	Metformin improved metabolic parameters without affecting remission
Gu et al., 2026 [28]	Higher CR with LNG-IUS plus metformin	Lower recurrence	Favorable reproductive outcomes	LNG-IUS plus metformin demonstrated superior overall outcomes

Abbreviations: CR, complete response; AEH, atypical endometrial hyperplasia; EEC, endometrioid endometrial cancer; PCOS, polycystic ovary syndrome; LNG-IUS, levonorgestrel-releasing intrauterine system; MA, megestrol acetate; MPA, medroxyprogesterone acetate; NR, not reported. Prog, progestin alone; Prog-Met, progestin plus metformin. Percentages are rounded as in the source reports. Recurrence denominators in the specified rows are patients achieving CR. Live-birth denominators differ and are specified by row; overall-cohort results must not be interpreted as metformin-specific effects.

## Data Availability

The authors declare that all related data are available from the corresponding author upon reasonable request.

## Supplementary Materials

The following supporting information can be downloaded at https://www.mdpi.com/article/10.3390/cancers18193141/s1, Table S1: PRISMA 2020 Checklist [48]; Table S2: Reported databases and illustrative search concepts.

## Abbreviations

	**Full Term**
AEH	Atypical Endometrial Hyperplasia
EIN	Endometrial Intraepithelial Neoplasia
EAH	Endometrial Atypical Hyperplasia
EEC	Endometrioid Endometrial Cancer
EC	Endometrial Cancer
EAC	Endometrial Adenocarcinoma
LNG-IUS	Levonorgestrel-Releasing Intrauterine System
MA	Megestrol Acetate
MPA	Medroxyprogesterone Acetate
CR	Complete Response
PCOS	Polycystic Ovary Syndrome
PMOS	Polyendocrine Metabolic Ovarian Syndrome
IVF	In Vitro Fertilization
ART	Assisted Reproductive Technology
BMI	Body Mass Index
AMPK	AMP-Activated Protein Kinase
mTOR	Mammalian Target of Rapamycin
mTORC1	Mammalian Target of Rapamycin Complex 1
PI3K	Phosphatidylinositol 3-Kinase
AKT	Protein Kinase B
IGF-1	Insulin-Like Growth Factor 1
IGF-1R	Insulin-Like Growth Factor 1 Receptor
ERα	Estrogen Receptor Alpha
S6K1	Ribosomal Protein S6 Kinase Beta-1
EIF4E	Eukaryotic Translation Initiation Factor 4E
PRISMA	Preferred Reporting Items for Systematic Reviews and Meta-Analyses
RCT	Randomized Controlled Trial (mentioned through study design context)
NR	Not Reported
