# Supplementary material for: Metformin as an Adjunct to Fertility-Sparing Treatment in Atypical Endometrial Hyperplasia and Early-Stage Endometrioid Endometrial Cancer: Narrative Review
Source: Cancers (Basel). 2026 Sep 28;18(19):3141. doi: 10.3390/cancers18193141 (PMC13664411; doi:10.3390/cancers18193141)
Supplement: Supplementary file 1 [file cancers-18-03141-s001.zip › cancers-4442278 Supplementary Table S2.pdf]

Supplementary Table S2. Reported databases and illustrative search concepts.

Reported search coverage: database inception through 31 May 2026. The database list and coverage reflect the manuscript's account; the original executable queries are unavailable for verification.

Database reported as searched	Illustrative concepts (not verified query terms)
PubMed/MEDLINE	Metformin; atypical endometrial hyperplasia/endometrial intraepithelial neoplasia; endometrial cancer; fertility-sparing or conservative treatment.
Embase	Metformin; atypical endometrial hyperplasia/endometrial intraepithelial neoplasia; endometrial cancer; fertility-sparing or conservative treatment.
Scopus	Metformin; atypical endometrial hyperplasia/endometrial intraepithelial neoplasia; endometrial cancer; fertility-sparing or conservative treatment.
Web of Science Core Collection	Metformin; atypical endometrial hyperplasia/endometrial intraepithelial neoplasia; endometrial cancer; fertility-sparing or conservative treatment.
Cochrane Library	Metformin; atypical endometrial hyperplasia/endometrial intraepithelial neoplasia; endometrial cancer; fertility-sparing or conservative treatment.

Note. The concepts are illustrative and do not reproduce the original executable queries. Original database-specific search histories and retrieval counts were unavailable. Boolean operators, controlled vocabulary and field tags therefore could not be retrospectively verified, and the low retrieval yield could not be explained from the available documentation. Only English-language publications were eligible; because non-English records were not documented separately, the contribution of this restriction to retrieval yield cannot be quantified.
